# Anticancer Activity of a Novel High Phenolic Sorghum Bran in Human Colon Cancer Cells

**DOI:** 10.1155/2020/2890536

**Published:** 2020-10-02

**Authors:** Seong-Ho Lee, Jihye Lee, Thomas Herald, Sarah Cox, Leela Noronha, Ramasamy Perumal, Hee-Seop Lee, Dmitriy Smolensky

**Affiliations:** ^1^Department of Nutrition and Food Science, College of Agriculture and Natural Resources, University of Maryland, College Park, MD 20742, USA; ^2^Agricultural Research Service, U.S. Department of Agriculture, Center for Grain and Animal Health Research, Manhattan, KS 66502, USA; ^3^Kansas State University, Agricultural Research Center, Hays, KS, USA

## Abstract

Human colon cancer is the third leading cause of mortality in the United States and worldwide. Chemoprevention using diet is widely accepted as a promising approach for cancer management. Numerous population studies indicate a negative correlation between the incidence of colon cancer and consumption of whole grains with a high content of bioactive phenolic compounds. In the current study, we evaluated the anticancer properties of a high phenolic sorghum bran extract prepared using 70% ethanol with 5% citric acid solvent at room temperature. A significant dose-dependent suppression of cell proliferation was observed in human colon cancer cells treated with the high phenolic sorghum bran extract. Apoptosis and S phase growth arrest were induced, while cell migration and invasion were inhibited by this treatment; these effects were accompanied by altered expression of apoptosis, cell cycle, and metastasis-regulating genes. We also found that the high phenolic sorghum bran extract stimulated DNA damage in association with induction of extracellular signal-regulated kinase (ERK) and c-Jun-NH_2_-terminal kinase (JNK) and subsequent expression of activating transcription factor 3 (ATF3). The present study expands our understanding of the potential use of high phenolic sorghum bran to prevent human colon cancer.

## 1. Introduction

In the United States, colon cancer ranks third in incidence and mortality among human cancers [1]. Approximately 70-75% of colon cancer is sporadic and therefore possibly preventable through avoiding exposure to a variety of environmental, pathological, and biological risk factors. Colon cancer is also highly associated with daily food intake patterns; low consumption of fiber-rich grains, vegetables, and fruits; and high consumption of red meat increase the risk of colon cancer [2]. Recently, plant-based diets have been receiving considerable attention in the area of chemoprevention. To date, thousands of phytochemicals have been identified, their potential as anticancer drugs tested in a broad range of doses, and their mechanisms of action investigated [3].

Sorghum (*Sorghum bicolor* L. Moench) is the fifth most commonly used cereal crop in the human diet worldwide. In addition to an abundance of major nutrients (starch, proteins, lipids, minerals, and vitamins), many sorghum varieties have a high content of bioactive compounds such as phenolic compounds [4]. However, the content of phenolic compounds is affected by several factors including the genotype, growing environment, and methods of processing and extraction [5, 6]. Recently, several research groups reported the anticancer activity for polyphenol-rich sorghum. Treatment of colon [7] and breast cancer cells [8] with sweet sorghum extracts led to a suppression of cell proliferation. One plausible explanation for the tumor suppressive activity of a variety of bioactive compounds is their natural antioxidative properties.

Recently, we screened 15 genetically diverse sorghum lines and identified a novel line with the high polyphenol content [5]. In a follow-up study, we aimed to identify the optimal extraction condition based on different solvents, pH, and temperature. As a result, we found that a 70% ethanol with 5% citric acid solvent was the most effective in terms of polyphenol content and antigrowth activity of colon cancer cells [9]. Therefore, we used this condition in our current study that was aimed at testing a novel high phenolic sorghum bran extract for anticancer properties and to elucidate its mechanism(s) of action in human colon cancer cells of different genetic backgrounds.

## 2. Materials and Methods

### 2.1. Preparation of High Phenolic Sorghum Bran Extract

An exotic high phenolic sorghum (accession PI570481) originally from Sudan was grown in Puerto Vallarta, Mexico, in a winter nursery during the 2016-2018 growing seasons. The high phenolic sorghum bran extract was prepared using a previously described method [9]. In brief, 10% bran *w*/*w*% was extracted using a 70% ethanol with 5% citric acid solvent at room temperature for 2 h, followed by storage at -20°C overnight. The sample was centrifuged at 1,000 g for 10 min, and the supernatant harvested and used as a crude extract.

### 2.2. Cell Lines and Antibodies

Human colon cancer cell lines (HCT15, SW480, HCT116, and HT-29) and noncolon cancer cell lines (3T3-L1, RAW264.7, and HUVEC) were purchased from American Type Culture Collection (Manassas, VA, USA). Antibodies for p21, p27, cyclin-dependent kinase 4 (CDK4), cyclin-dependent kinase 6 (CDK6), *β*-catenin, matrix metalloproteinase 9 (MMP9), p-H2AX, phosphorylated extra cellular signal-regulated kinase (p-ERK), phosphorylated c-Jun-NH_2_-terminal kinase (p-JNK), poly(ADP-ribose) polymerase (PARP), caspase-3, and Bcl-xL were purchased from Cell Signaling (Danvers, MA, USA). Antibodies for actin, Bak, cyclin D1, vascular endothelial growth factor (VEGF), and activating transcription factor 3 (ATF3) were purchased from Santa Cruz Biotechnology (Santa Cruz, CA, USA).

### 2.3. Cell Culture and Treatment

Human colon cancer cells were cultured at 37°C under a humidified atmosphere of 5% CO_2_ using Dulbecco's modified Eagle medium (DMEM), McCoy's medium, and RPMI medium supplemented with 10% fetal bovine serum (FBS), 100 U/ml penicillin, and 100 *μ*g/ml streptomycin. The high phenolic sorghum bran extract was diluted using a 70% ethanol with 5% citric acid solvent (vehicle) in culture medium to make final concentrations of 0, 0.625, 1.25, 2.5, 5.0, and 10.0 mg/ml (*w*/*v*). All chemicals and cell culture media were purchased from Fisher Scientific (Pittsburg, PA), unless otherwise specified.

### 2.4. Cell Proliferation Assay

Cell proliferation was measured using the MTS assay as described previously [5]. Briefly, four types of human colon cancer cells were seeded onto a 96-well culture plate at 2 × 10^4^ cells/well and incubated for 48 h to allow complete attachment. Then, the cells were treated with 0, 0.625, 1.25, 5.0, and 10.0 mg/ml of the high phenolic sorghum bran extract in complete medium for 24, 48, and 72 h. A 70% ethanol with 5% citric acid solution was used as the vehicle. After washing with phosphate-buffered saline (PBS), 20% MTS solution was added to each well, the cells were incubated at 37°C for 30 min, and absorbance was measured at 490 nm using a BioTek (Winooski, Vermont) H4 Plate Reader. Cell viability was measured using MTT assay as described previously [10].

### 2.5. Apoptosis Assay

Apoptosis was assessed using the Alexa Fluor 488 Annexin V/Dead Cell Apoptosis Kit (Fisher Scientific) according to the manufacturer's instructions as described earlier [5]. Briefly, the cells were treated with 0, 1.25, and 2.5 mg/ml of sorghum extract for 18 h in serum-free medium. Then both, floating and attached cells were collected by trypsinization for 10 min and subsequent centrifugation at 600 g for 10 min. After washing with PBS twice, the cells were suspended in Annexin V/propidium iodide staining buffer. Florescence was measured on Alexa Fluor 488 and Texas Red channels using a BD LSRFortessa™ system (BD Biosciences, San Jose, CA). Results are reported as mean ± SE from four independent experiments.

### 2.6. Cell Cycle Analysis

Cell cycle distribution was analyzed as described earlier [11]. In brief, the cells were treated with 0, 1.25, and 2.5 mg/ml of sorghum extract for 48 h in complete medium containing 10% FBS. Then, the cells were harvested by trypsinization for 10 min and fixed in 70% ethanol solution at -20°C. After centrifugation, the cells were washed with PBS followed by PI/RNAse staining buffer (BD Biosciences, San Jose, CA). After centrifugation, the cells were resuspended and incubated with PI/RNase staining buffer for 15 min. Fluorescence was measured for 10,000 gated events on the Texas Red channel using a BD LSRFortessa™ system (BD Biosciences, San Jose, CA). Results are reported as mean ± SE from four independent experiments.

### 2.7. Cell Migration Assay

Cells were plated onto a 6-well plate and incubated overnight. On the following day, the scratch was created using a P200 pipette tip. After washing to remove debris, the cells were incubated with fresh complete medium containing 0, 1.25, and 2.5 mg/ml high phenolic sorghum bran extract. After incubation for 24 h (SW480 and HCT116) or 48 h (HCT15), the cells were photographed under a phase-contrast microscope (Nikon ECLIPS Ti, Melville, NY, USA).

### 2.8. Cell Invasion Assay

Cell invasion was measured using Matrigel according to the manufacturer's instructions (Corning, MA, USA). Briefly, cells were plated on top of the filter membrane in a Transwell insert and incubated at 37°C and 5% CO_2_ for 10 min to allow them to settle. The high phenolic sorghum bran extract was then added to the bottom well, and the cells were incubated for 18 h. After removal of the Transwell insert from the plate, 70% ethanol was added for 10 min for cell fixation and crystal violet was added to stain the invading colony.

### 2.9. Tumor Formation Assay

Tumor formation was analyzed using Cultrex In Vitro Angiogenesis Assay Kit (Cat#: ECM625, Milipore, Burlington, MA, USA) according to the manufacturer's instructions. Human umbilical vein endothelial cells (HUVEC) were cultured in Endothelial Cell Growth Base Media (Cat#: CCM027; Minneapolis, MN, USA) and plated on ECMatrix™-coated 96-well plate and incubated with with 0, 1.25, and 2.5 mg/ml high phenolic sorghum bran extract for 6 h. Vascular networks were captured under a phase-contrast microscope (Nikon ECLIPS Ti, Melville, NY, USA) and analyzed with ImageJ's tool “Angiogenesis Analyzer” to quantify the numbers of tubes, junctions, and branches and total tube length. (Supplementary Figure 2).

### 2.10. Transient Transfection and Luciferase Assay

DNA constructs with reporter genes (generous gift from Dr. Seung Joon Baek, University of Tennessee, Knoxville) were transiently transfected into cells using a Polyjet DNA transfection reagent (SignaGen Laboratories, Ijamsville, MD, USA) according to the manufacturer's instructions. Cells (2 × 10^5^ cells/well) were plated onto 24-well dishes in triplicate and incubated overnight. On the following day, plasmid mixtures containing 1 *μ*g of reporter plasmid and 0.1 *μ*g of *pRL-null* were transfected into the cells for 24 h. The transfected cells were then treated with the indicated concentrations of the high phenolic sorghum bran extract for 18 h. Subsequently, cell lysates were extracted with luciferase lysis buffer and luciferase activity was measured and normalized to the *pRL-null* luciferase activity using a dual-luciferase assay kit (Promega, Madison, WI, USA).

### 2.11. SDS-PAGE and Western Blot

Cells were washed with ice-cold PBS, and cell lysates were extracted using radioimmunoprecipitation assay (RIPA) buffer supplemented with a cocktail of protease and phosphatase inhibitors. After centrifugation at 12,000 g at 4°C for 10 min, the supernatant was collected and protein concentration was determined using the bicinchoninic acid protein assay (Pierce, Rockford, IL, USA). Thirty microgram of protein was separated by SDS-PAGE and transferred onto nitrocellulose membranes (Osmonics, Minnetonka, MN, USA). After blocking with 5% nonfat milk in Tris-buffered saline containing 0.05% Tween-20 for 1 h, the membranes were probed with primary antibodies with 1 : 500-1 : 1000 dilution rate at 4°C overnight. The antibodies for PARP (#9542), caspase-3 (#9664), Bcl-xL (#2764), actin (#5125), p21 (#2947), p27 (#3686), CDK6 (#3136), CDK4 (#12790), *β*-catenin (#9582), MMP9 (#13667), pH2AX (#9718), p-ERK (#9101), and p-ERK (#4668) were purchased from Cell Signaling Technology (Beverly, MA, USA). The antibodies for Bak (#832), cyclin D1 (#717), VEGF (#567), and ATF3 (#188) were purchased from Santa Cruz Biotechnology. The membranes were incubated with horseradish peroxidase-conjugated immunoglobulin G for 1 h. Chemiluminescence was detected with the Pierce ECL Western Blotting Substrate (Thermo Scientific, Waltham, MA, USA) and visualized by the Chemidoc MP Imaging System (Bio-Rad, Hercules, CA, USA).

### 2.12. Measurement of Reactive Oxygen Species (ROS)

ROS were measured using the OxiSelect Intracellular ROS Assay Kit (Cell Biolabs, Atlanta, GA, USA) based on the manufacturer's instructions. Briefly, 5 × 10^4^ cells were plated onto a 96-well plate and incubated at 37°C overnight. A specific ROS-detecting fluorescent dye, 2′,7′,-dichlorodihydrofluorescein diacetate (DCFH-DA) was added for 1 h. Cells were washed with PBS twice, medium and 2X cell lysis buffer were added, and fluorescence was measured using the BioTek Synergy HT (Em: 480 nm/Ex: 530 nm). Fluorescence intensity was normalized using the MTT assay.

### 2.13. Statistical Analysis

Statistical analyses were performed with ANOVA followed by Tukey's test for multiple comparisons (cell proliferation, apoptosis, and cell cycle) or unpaired Student's *t*-test (western blot and luciferase activity). Data are expressed as mean ± SE or mean ± SD as indicated in the figure legends. Differences were considered significant at *P* < 0.05.

## 3. Results

### 3.1. High Phenolic Sorghum Bran Extract Suppressed Proliferation of Human Colon Cancer Cells

**Figure 1:**
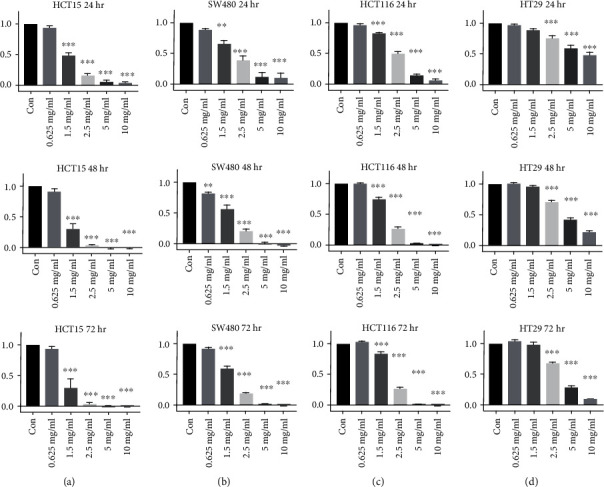
Effect of high phenolic sorghum bran extract on proliferation of multiple human colon cancer cell lines. HCT15, SW480, HCT116, and HT-29 (a-d) cells were plated onto 96-well culture dishes and treated with 0, 0.625, 1.25, 2.5, 5.0, and 10.0 mg/ml of high phenolic sorghum bran extract for 0, 24, 48, and 72 h. The MTS assay was performed to measure cell proliferation. Values are mean ± SE (*n* = 3). Significance is indicated by ^∗^*P* < 0.05, ^∗∗^*P* < 0.01, and ^∗∗∗^*P* < 0.001 (difference between vehicle and treatment).

Previously, we reported the antiproliferative activity of a high phenolic sorghum bran extract prepared using 50% (*v*/*v*) ethanol in human cancer cells [5] and development of a more efficient method using 70% ethanol with 5% citric acid solvent [9]. To further our understanding of the anticancer activity of the high phenolic sorghum bran extracted with the 70% ethanol with 5% citric acid solvent, we measured cell proliferation in four human colon cancer cell lines (HCT15, SW480, HCT116, and HT-29) treated with different concentrations of the extract. Our results showed that treatment with the extract suppressed cell proliferation in a dose- and time-dependent manner in all four cell lines (Figures 1(a)–1(d)). The highest growth inhibitory activity was observed in HCT15 and SW480 cells, while HT-29 cells were resistant to the same high dose (10 mg/ml). Therefore, we selected HCT15, SW480, and HCT116 for further studies of the anticancer properties and mechanisms of action of the high phenolic sorghum bran extract. To examine if the used concentrations of high phenolic sorghum bran extract may cause cytotoxicity, we measured viability using two non-colorectal cancer cell lines (normal 3T3-L1 and macrophage like RAW264.7). No toxicity was observed in the cells treated with 0-10 mg/ml of high phenolic sorghum bran extracts (Supplementary Figure 1).

### 3.2. High Phenolic Sorghum Bran Extract Induced Apoptosis of Human Colon Cancer Cells

**Figure 2:**
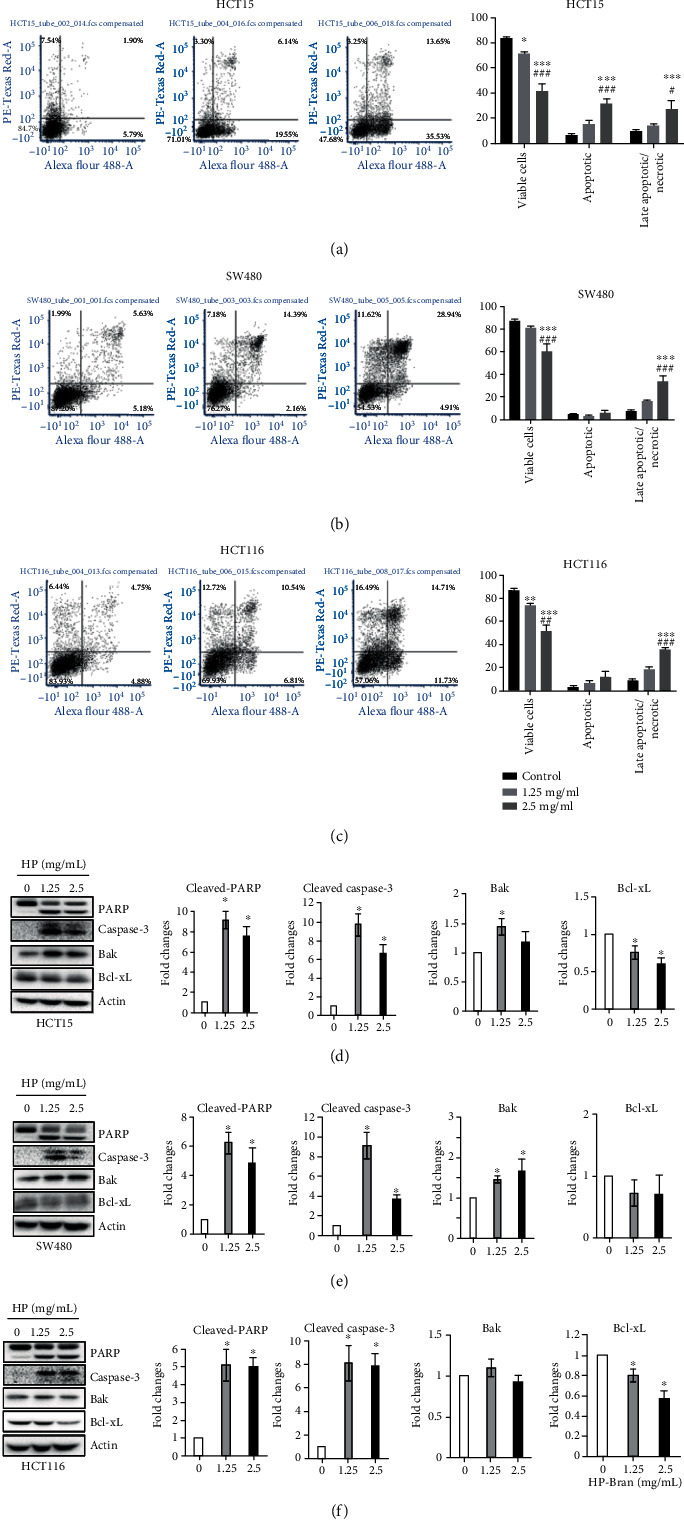
Effect of high phenolic sorghum bran extract on apoptosis of multiple human colon cancer cell lines. (a–c) HCT15, SW480, and HCT116 cells were treated with 0, 1.25, and 2.5 mg/ml of high phenolic sorghum bran extract for 18 h, and apoptosis was measured using the Alexa Fluor 488 Annexin V/Dead Cell Apoptosis Kit. Values are mean ± SE (*n* = 3). (d–f) HCT15, SW480, and HCT116 cells were treated with 0, 1.25, and 2.5 mg/ml of high phenolic sorghum bran extract (HP) for 18 h, and western blot was performed to measure the expression of indicated proteins. Values are mean ± SD (*n* = 3). Significance is indicated by ^∗^*P* < 0.05, ^∗∗^*P* < 0.01, and ^∗∗∗^*P* < 0.001 (difference between vehicle and treatment); ^#^*P* < 0.05, ^##^*P* < 0.01, and ^###^*P* < 0.001 (difference between 1.25 mg/ml and 2.5 mg/ml).

Apoptosis is defined as a programed cell death induced by several internal and external signals including DNA damage, cell stress, and proapoptotic cytokines. To explore if growth inhibition by the high phenolic sorghum bran extract was associated with increased apoptosis, we analyzed the percentages of apoptotic cells from cells treated with 0, 1.25, and 2.5 mg/ml of the high phenolic sorghum bran extract. The results showed induction of apoptosis (percent of apoptotic and late apoptotic/necrotic cells combined) from 16.6% (control) to 28.9% (1.25 mg/ml) and 58.9% (2.5 mg/ml) in HCT15 cells treated with the high phenolic sorghum bran extract (Figure 2(a)). Similar results were obtained from SW480 cells with an induction of apoptosis from 12.4% (control) to 19.1% (1.25 mg/ml) and 39.6% (2.5 mg/ml), and from HCT116 cells with an induction of apoptosis from 12.8% (control) to 25.6% (1.25 mg/ml) and 48.4% (2.5 mg/ml) (Figures 2(b) and 2(c)). Next, we measured the expression of apoptosis-regulating genes. As shown in Figures 2(d) and 2(e), treatment with the high phenolic sorghum bran extract dramatically induced expression of the apoptosis markers, cleaved PARP, and caspase-3. In addition, we observed upregulation of the proapoptotic marker Bak and downregulation of the antiapoptotic marker Bcl-xL, indicating an induction of the mitochondria-dependent apoptotic pathway.

### 3.3. High Phenolic Sorghum Bran Extract Induced Growth Arrest at S Phase in Human Colon Cancer Cells

**Figure 3:**
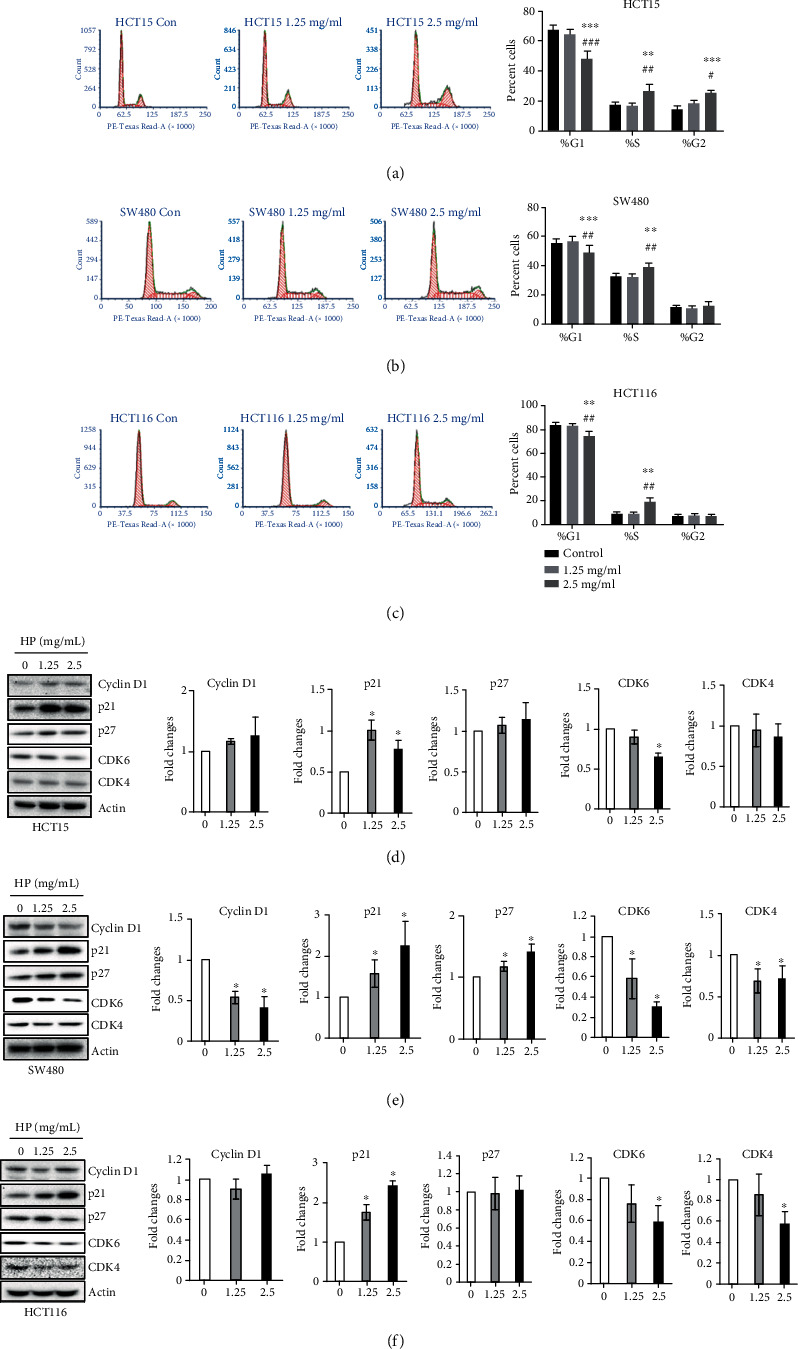
Effect of high phenolic sorghum bran extract on cell cycle distribution of multiple human colon cancer cell lines. (a–c) HCT15, SW480, and HCT116 cells were treated with 0, 1.25, and 2.5 mg/ml of high phenolic sorghum bran extract for 18 h, and the cell cycle distribution was measured by FACS analysis. Values are mean ± SE (*n* = 3). (d–f) HCT15, SW480, and HCT116 cells were treated with 0, 1.25, and 2.5 mg/ml of high phenolic sorghum bran extract (HP) for 18 h, and western blot was performed to measure the expression of indicated proteins. Values are mean ± SD (*n* = 3). Significance is indicated by ^∗^*P* < 0.05, ^∗∗^*P* < 0.01, and ^∗∗∗^*P* < 0.001 (difference between vehicle and treatment); ^#^*P* < 0.05, ^##^*P* < 0.05, and ^###^*P* < 0.05 (difference between 1.25 mg/ml and 2.5 mg/ml).

The cell cycle is a process where cells replicate their DNA and distribute duplicated chromosomes with high accuracy to their daughter cells for inheritance. The cell cycle is regulated by the coordination of cyclins, CDKs, and CDK inhibitors. The DNA checkpoint is a proofreading mechanism for ensuring the fidelity of the cell cycle. In particular, the growth arrest machinery could be a molecular target for cancer treatment. In order to test if growth inhibition by the high phenolic sorghum bran extract was associated with growth arrest, we measured changes in cell distribution in the cell cycle phases. The results indicate that treatment with the high phenolic sorghum bran extract led to a significant increase in the number of cells in the S phase with a concomitant decrease in the number of cells in G1 phase, indicating an S phase arrest. We also measured the expression of cell cycle-regulating genes by western blot. As shown in Figures 3(d) and 3(e), treatment with the high phenolic sorghum bran extract slightly reduced the expression of cyclin D1, CDK4, and CDK6 and increased the expression of p21 and p27.

### 3.4. High Phenolic Sorghum Bran Extract Suppressed Migration and Invasion of Human Colon Cancer Cells

**Figure 4:**
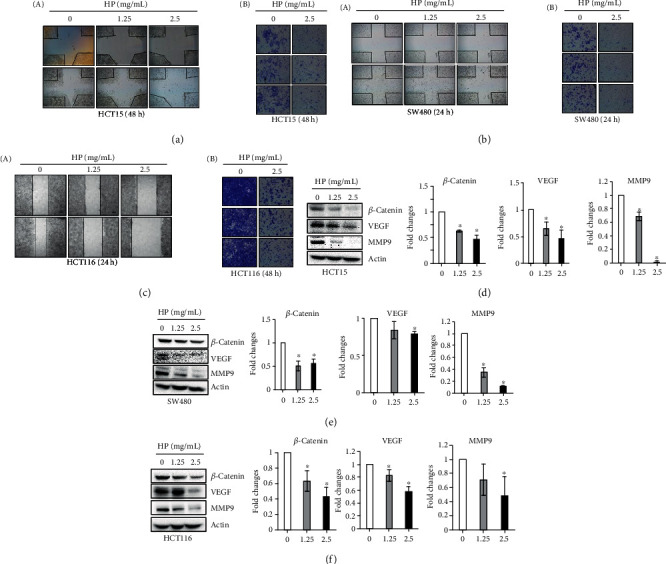
Effect of high phenolic sorghum bran extract on *in vitro* metastatic activity of multiple human colon cancer cell lines. (a–c) HCT15, SW480, and HCT116 cells were treated with 0, 1.25, and 2.5 mg/ml of high phenolic sorghum bran extract (HP) for indicated time and cell migration (left) and invasion (right) were measured using a wound healing assay and Matrigel, respectively. (d–f) HCT15, SW480, and HCT116 cells were treated with 0, 1.25, and 2.5 mg/ml of HP for 18 h, and western blot was performed to measure expression of indicated proteins. Values are mean ± SD (*n* = 3). ^∗^ indicates significant differences at *P* < 0.05 (between treatment and control).

Metastasis is a complex process where original tumors spread to other parts of the body through the circulatory system. The initial step of metastasis is migration and invasion. The invasion of cancer cells is characterized by cell adhesion to the extracellular matrix (ECM) and subsequent ECM degradation. Therefore, in order to examine if the high phenolic sorghum bran extract possesses an antimetastatic activity, we performed *in vitro* cell migration and invasion assays using three invasive human colon adenocarcinoma cell lines. Our results (Figures 4(a)–4(c)) indicate a significant inhibition of migration and invasion. To investigate the relevant mechanism, we compared the expression of metastasis-regulating genes. We observed a significant downregulation of the expression of *β*-catenin, which plays a key role in metastasis. Since ECM degradation is mediated by MMPs, we also measured the expression of MMP9 and found a significant downregulation (Figures 4(d) and 4(e)). The invasion of cancer cells leads to the formation of new blood vessels; therefore, we measured the expression of the angiogenesis marker VEGF that is synthesized and secreted by invasive cancer cells. The result showed suppressed expression of VEGF in the cells treated with the high phenolic sorghum bran extract (Figures 4(d) and 4(e)). However, *in vitro* tube formation was not changed by sorghum bran extract (Supplementary Figure 2).

### 3.5. High Phenolic Sorghum Bran Extract Induced DNA Damage and MAPK Activation in Human Colon Cancer Cells

**Figure 5:**
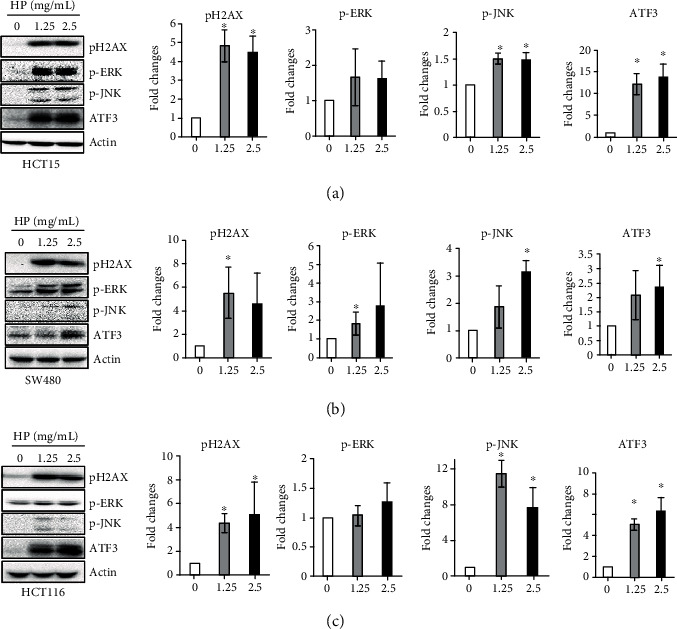
Effect of high phenolic sorghum bran extract on expression of DNA damage-associated genes in multiple human colon cancer cell lines. (a–c) HCT15, SW480, and HCT116 cells were treated with 0, 1.25, and 2.5 mg/ml of high phenolic sorghum bran extract (HP) for 18 h. Western blot was performed to measure the expression of indicated proteins. Values are mean ± SD (*n* = 3). Significant differences at ^∗^*P* < 0.05 (between treatment and control).

To determine if the high phenolic sorghum bran extract induces DNA damage, we measured the phosphorylation of H2AX, a marker of DNA double-strand breaks and DNA damage. As shown in Figure 5, the high phenolic sorghum bran extract significantly increased the phosphorylation of H2AX in a dose-dependent manner in HCT15, SW480, and HCT116 cells. Since DNA damage-induced apoptosis is mediated by ERK [12] and JNK [13], we also measured the phosphorylation of these proteins. We found that phosphorylation of both ERK and JNK in the cells was induced by treatment with the extract (Figure 5). We and others recently identified ATF3 as a target of DNA damage [14, 15] that mediates the apoptosis induced by many anticancer compounds [16–18]. Therefore, we further explored if the high phenolic sorghum bran extract-induced DNA damage was associated with overexpression of ATF3. We found that ATF3 expression was highly induced in all three human colon cancer cell lines (Figure 5).

## 4. Discussion

The significance of plant-based diets in the prevention and management of cancer has received much attention in the past decade. There is a growing body of evidence from *in vitro* and *in vivo* studies indicating that sorghum consumption can provide diverse benefits in the prevention of human chronic diseases including cancer [19–22]. The plausible explanation for these health benefits is the high content of phytochemicals in sorghum. In particular, polyphenols possess strong antioxidative activities. The most abundant phytochemicals in sorghum are polyphenols, which can be classified into several groups including flavonoids, phenolic acids, and tannins. Phenolic acids are major components in sorghum whereas tannins are detected in only a few species [23].

Recently, we identified a novel black sorghum genotype that contains a high amount of polyphenols [5]. We optimized its extraction conditions based on solvent, pH, and temperature in order to maximize the yield of polyphenols. In this study, we used a high phenolic sorghum bran extract prepared from a 70% ethanol with 5% citric acid solvent at room temperature. Here, for the first time, we demonstrate anticancer properties of the polyphenol-abundant sorghum bran in a human colon cancer model.

The growth inhibitory activity of the high phenolic sorghum bran extract was observed in four different human colon adenocarcinoma cell lines (HCT15, SW480, HCT116, and HT-29), although each cell line showed different responses to the same high dose. Our data indicate that growth inhibition is a common outcome for colon cancer cells treated with the high phenolic sorghum bran extract. In addition, these four human colon cancer cell lines have different genetic backgrounds. For example, SW480 and HT-29 have mutated p53, while HCT15 and HCT116 have wild-type p53, indicating that the anticancer activity of the high phenolic sorghum bran extract is p53 independent. In fact, we observed that p53 expression was unaltered by treatment with the high phenolic sorghum bran extract (data not shown).

One of the interesting findings in this study is that the high phenolic sorghum bran extract showed strong antimetastatic activity in invasive colon adenocarcinoma cells. The suppressive activity on cell migration and invasion was accompanied by the downregulation of MMP9 and VEGF proteins. These results prompt the question of whether the high phenolic sorghum bran extract could prevent the spread of colon cancer in advanced stages. Further *in vivo* studies using orthotopic animal models would be required to examine the effects of the high phenolic sorghum bran extract on colon cancer metastasis to the liver and lung. While the extract did not significantly inhibit tube formation by HUVEC cells, this result can be attributed to the fact that high phenolic sorghum bran extract lowered the expression of VEGF in colorectal cancer cells, but the tube formation assay utilizes exogenous VEGF protein. These results further indicate that these polyphenols have potential inhibitory effects on the tumor while not having deleterious effects in normal tissues.

Our data showed that the high phenolic sorghum bran extract increased the expression of cleaved caspase-3 and cleaved PARP, which belong to the caspase family and are involved in the downstream events of mitochondria-mediated apoptosis. Our results also showed that the high phenolic sorghum bran extract could arrest the cell cycle at S phase.

Another interesting finding in the current study is that the high phenolic sorghum bran extract increased DNA damage, which could be a common consequence of anticancer and antiapoptotic activities. We also confirmed the activation of ERK and JNK, which are downstream target kinases of DNA damage-induced apoptosis [12, 13]. Recently, we proposed DNA damage-stimulated overexpression of ATF3 as a potential anticancer mechanism [14]. Since treatment with the high phenolic sorghum bran extract stimulated phosphorylation and expression of DNA damage-associated genes and ATF3, we speculate that the sorghum bran extract exerts its anticancer effects via the same mechanism proposed for other anticancer drug such as tolfenamic acid [14].

The transcription factor NF-*κ*B mediates anticancer agent-induced apoptosis [24–27] in several types of cancer cell lines and mediates anticancer drug-induced ATF3 expression in colon cancer cells [14]. Thus, we measured the transcriptional activity of NF-*κ*B using a reporter plasmid. We found that transcriptional activity was induced in three colon cancer cell lines treated with the high phenolic sorghum bran extract, although the responsiveness varies substantially depending on the cell type (Supplementary Figure 3). Since the anticancer activity of polyphenols was also associated with ROS generation, we measured intracellular ROS levels after staining the cells with DCFH-DA. Treatment with 1.25 and 2.5 mg/ml of the high phenolic sorghum extract resulted in a significant ROS increase in HCT15 cells but not SW480 and HCT116 cells (Supplementary Figure 4). These results indicate that increased DNA damage and resultant apoptosis might be ROS dependent or independent depending on the cell type. In fact, ROS-independent induction of DNA damage and apoptosis has been observed in several other studies [28].

**Figure 6:**
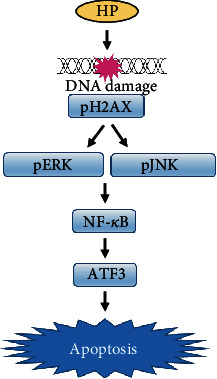
Proposed mechanism of anticancer activity of high phenolic sorghum bran extract. HP: high phenolic sorghum bran extract.

In summary, the dietary consumption of high phenolic sorghum bran offers an alternative and safe colon cancer preventive measure that targets multiple molecular pathways. The data presented here elucidates some of the mechanisms involved, including activation of DNA damage, MAPKs, and NF-*κ*B transcriptional activity. The resultant activation of this axis leads to an increase of the proapoptotic protein, ATF3 (Figure 6).

## Data Availability

All data is maintained within the manuscript. Request for raw data can be made.

## Supplementary Materials

Supplementary Materials The following are available online at http://www.mdpi.com/xxx/s1. Figure S1: effect of high phenolic sorghum bran extract on viability of normal cells (3T3-L1 and RAW264.7). Figure S2: effect of high phenolic sorghum bran extract on *in vitro* tube formation. Figure S3: effect of high phenolic sorghum bran extract on the transcriptional activity of NF-*κ*B in multiple human colon cancer cell lines, Figure S4: effect of high phenolic sorghum bran extract on reactive oxygen species (ROS) production in multiple human colon cancer cell lines.
