# Supplementary figures and images for: Anticancer Activity of a Novel High Phenolic Sorghum Bran in Human Colon Cancer Cells
Source: Oxid Med Cell Longev. 2020 Oct 2;2020:2890536. doi: 10.1155/2020/2890536 (PMC7556115; doi:10.1155/2020/2890536)

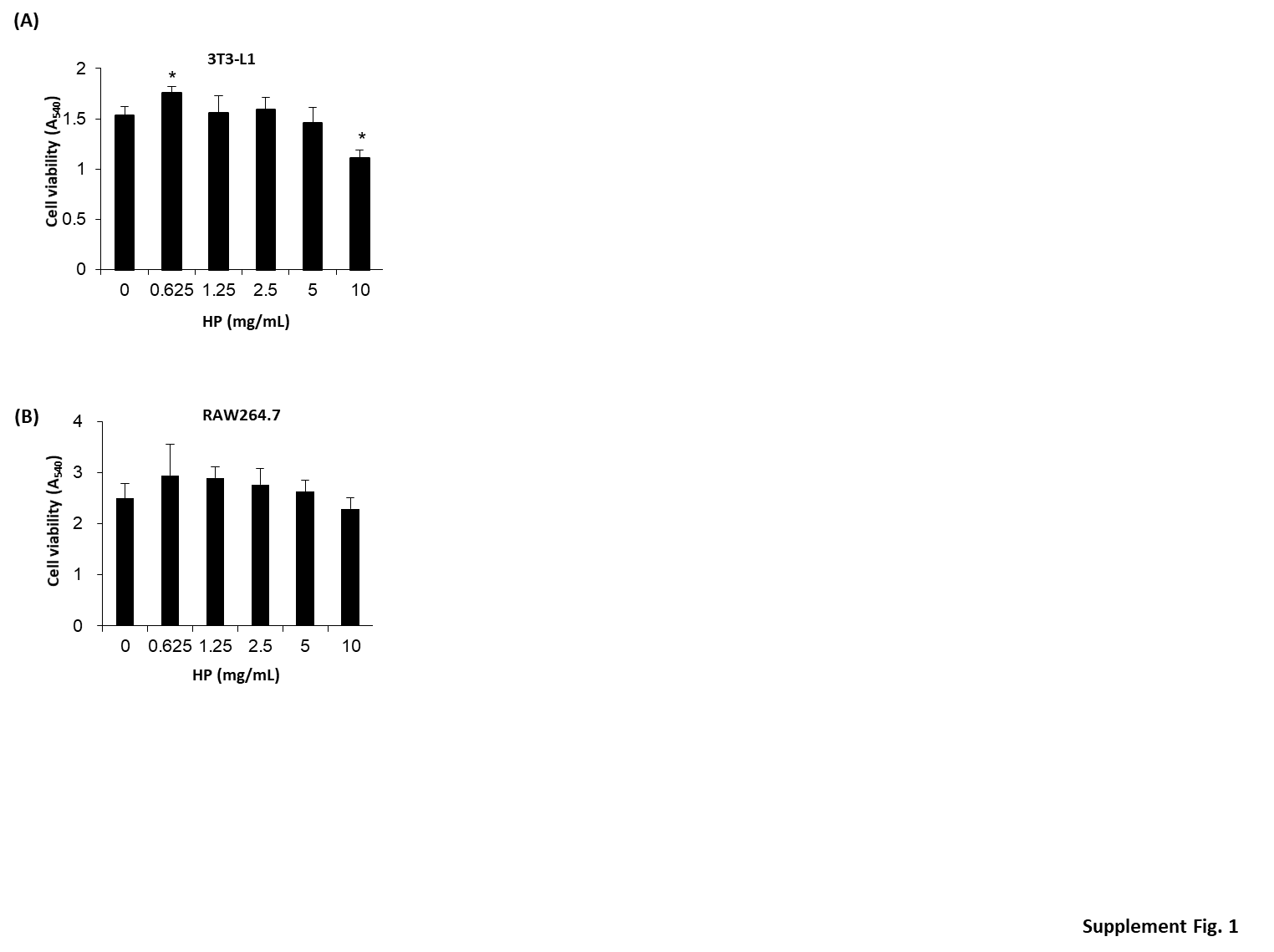


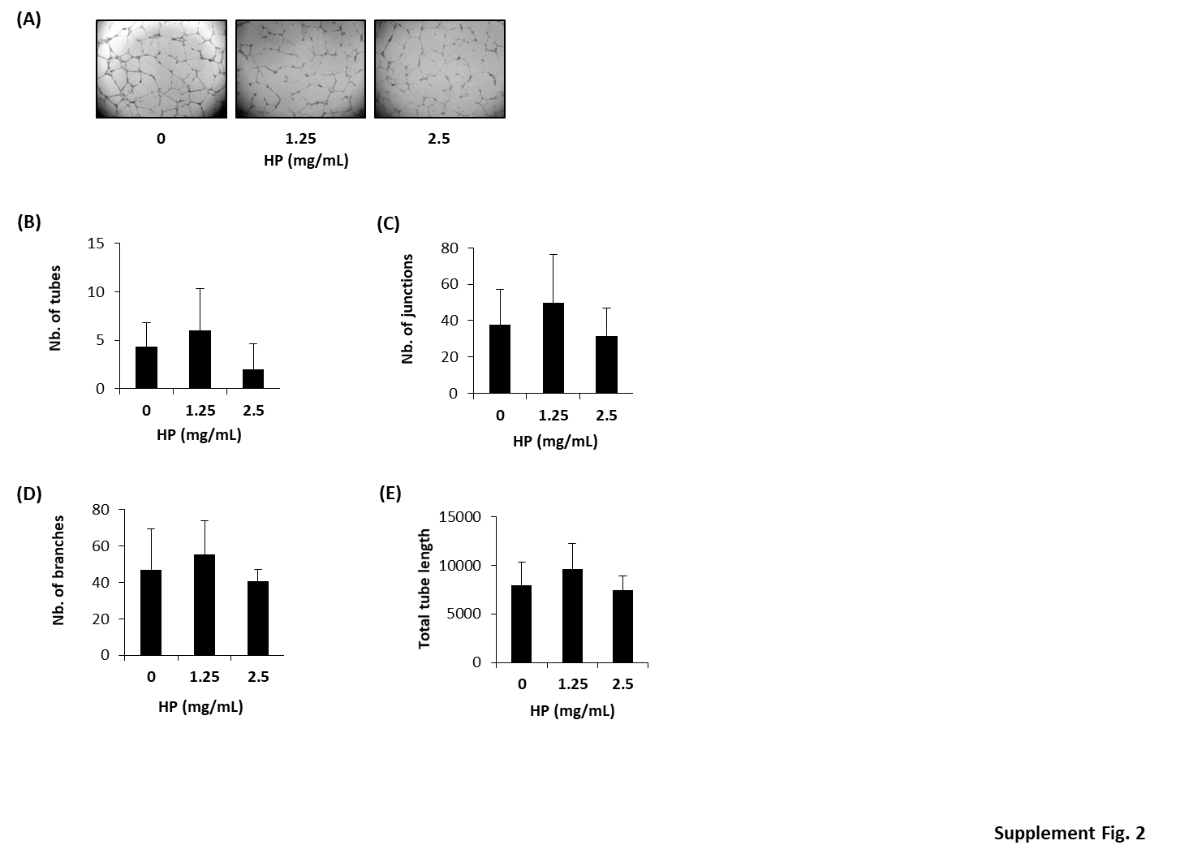


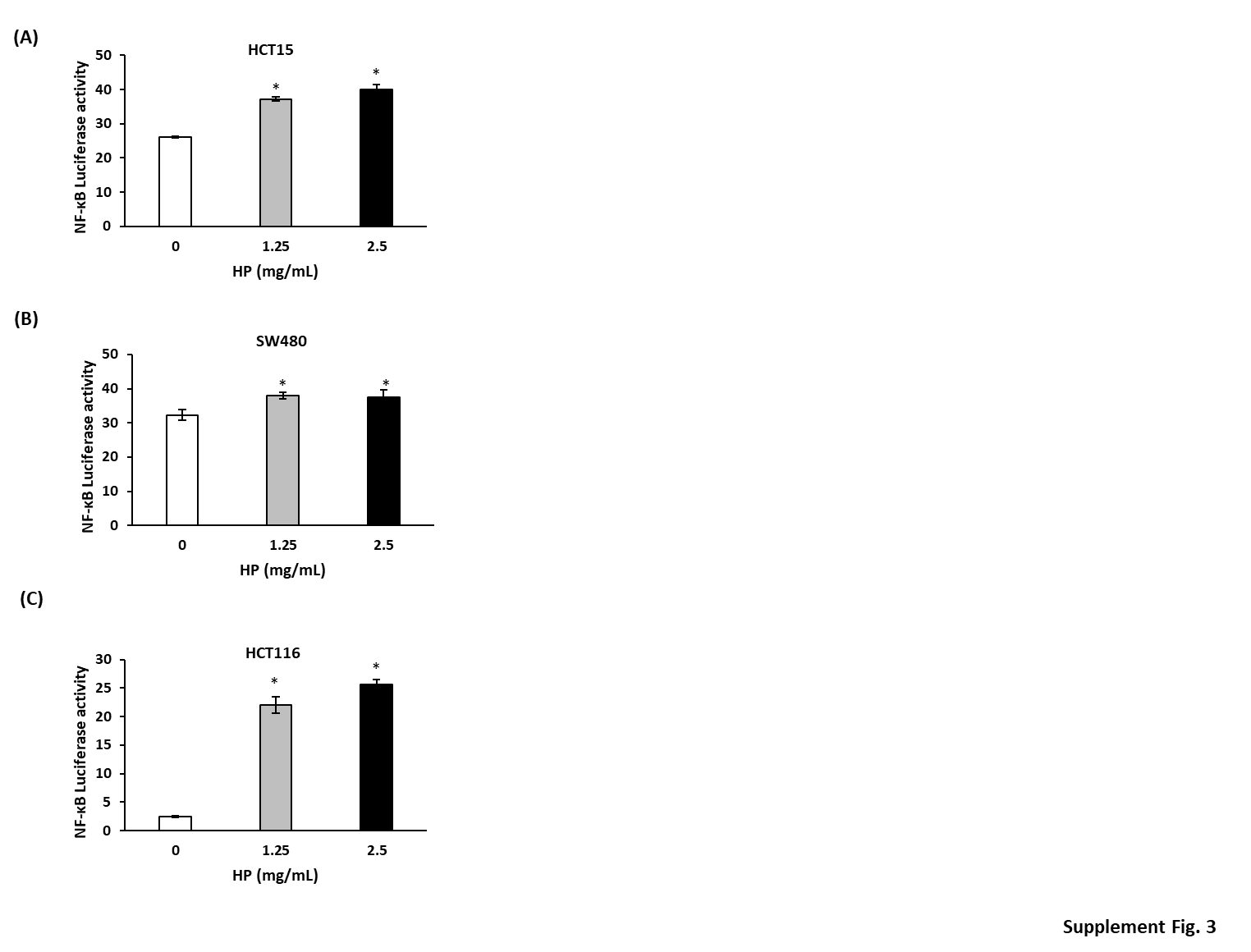


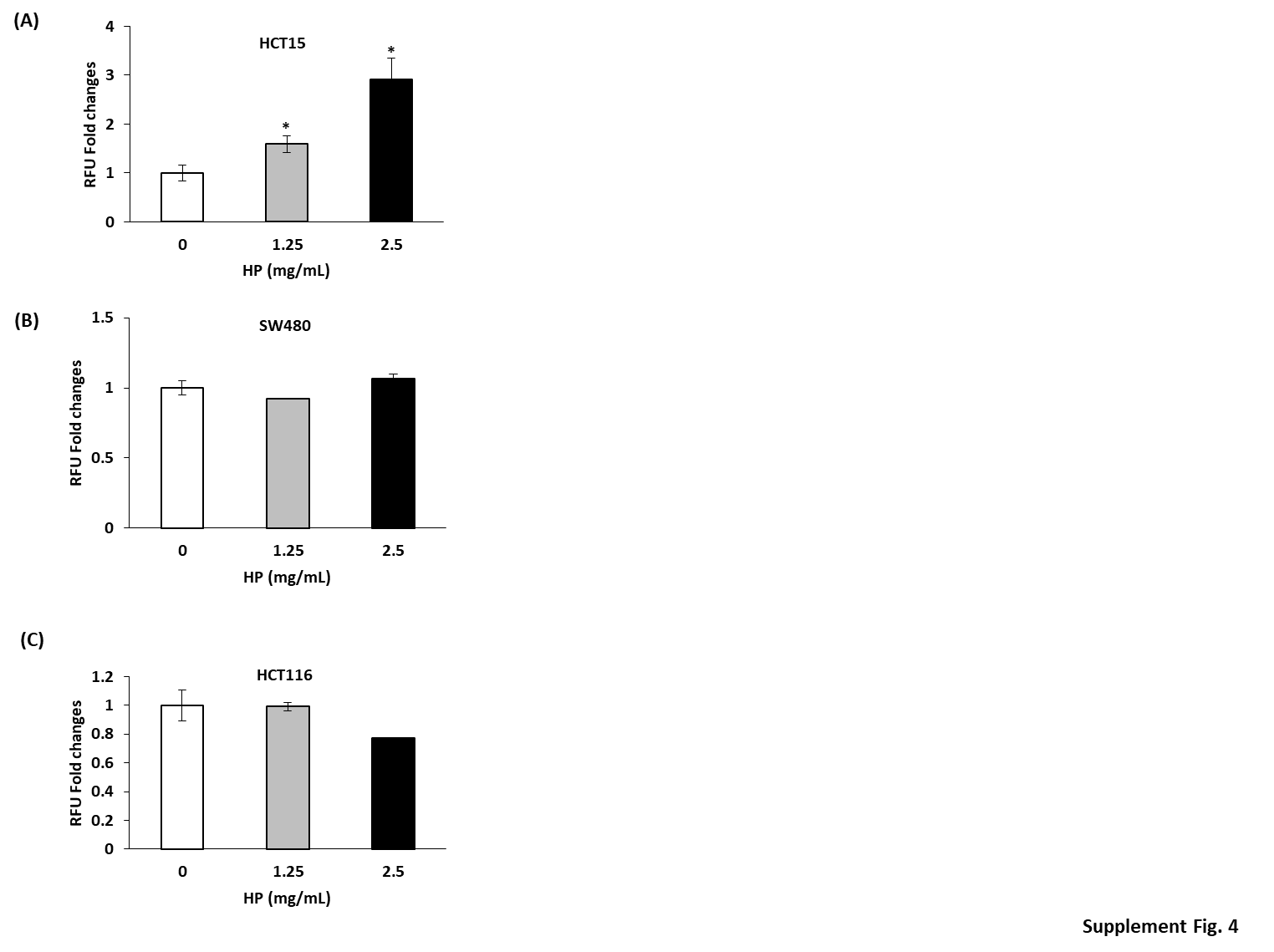

Supplement: Supplementary Materials — The following are available online at http://www.mdpi.com/xxx/s1. Figure S1: effect of high phenolic sorghum bran extract on viability of normal cells (3T3-L1 and RAW264.7). Figure S2: effect of high phenolic sorghum bran extract on in vitro tube formation. Figure S3: effect of high phenolic sorghum bran extract on the transcriptional activity of NF-κB in multiple human colon cancer cell lines, Figure S4: effect of high phenolic sorghum bran extract on reactive oxygen species (ROS) production in multiple human colon cancer cell lines. [file 2890536.f1.docx]
